# Vision–Language Models for Transmission Line Fault Detection: A New Approach for Grid Reliability and Optimization

**DOI:** 10.3390/jimaging12030106

**Published:** 2026-02-28

**Authors:** Runle Yu, Lihao Mai, Yang Weng, Qiushi Cui, Guochang Xu, Pengliang Ren

**Affiliations:** 1 School of Electrical Engineering, Chongqing University, Chongqing 400044, China; 2 School of Electrical, Computer and Energy Engineering, Arizona State University, 551 East Tyler Mall, Tempe, AZ 85281, USA; 3 State Grid Henan Electric Power Company, Zhengzhou 450052, China; 4 Henan Jiuyu EPRI Electric Power Technology Co., Ltd., Zhengzhou 450052, China

**Keywords:** transmission corridor, fault detection, vision language model, subclass aware fusion, geometry preserving normalization, geo prior, calibration

## Abstract

Reliable fault detection along transmission corridors is essential for preventing small defects from developing into long outages and costly emergency operations. This study aims to improve the field reliability of an open vocabulary vision language backbone without retraining the large model in an end-to-end manner. The work focuses on four operational fault classes in multi-region corridor imagery collected during routine inspections and uses a Florence-2 vision language model as the base recognizer. On top of this backbone, three domain-specific components are introduced. A subclass-aware fusion scheme keeps probability mass within the active parent concept so that insulator icing and conductor icing produce stable, action-oriented decisions. A Power-Line Focus Then Crop normalization uses an attention-guided corridor window together with isotropic resizing so that thin conductors and small fittings remain visible in the processed image. A corridor geo prior reduces scores as the distance from the mapped centerline increases and in this way suppresses detections that lie outside the corridor. All methods are evaluated under a shared preprocessing and scoring pipeline in training-free and parameter-efficient tuning modes. Experiments on unseen regions show higher accuracy for skinny and low-contrast faults, fewer false alarms outside the right-of-way, and improved score calibration in the confidence range used for triage, while keeping throughput and memory usage suitable for unmanned aerial vehicles and substation edge devices.

## 1. Introduction

Electric power transmission corridors are inspected to prevent small defects from developing into long outages and costly emergency operations [1,2,3]. Traditional right-of-way patrols by ground crews and helicopters are effective, but they are expensive, sensitive to weather, and difficult to scale as networks expand and extreme events become more frequent [4,5,6]. In recent years, high-resolution sensing on unmanned aerial vehicles together with modern computer vision has changed how utilities observe conductors, insulators, fittings, and surrounding vegetation [7,8,9]. At the same time, vision language models trained on large image and text corpora provide open vocabulary recognition. Operators can search for faults using descriptive prompts instead of a fixed label list, which is attractive for evolving assets and rare failure modes [10,11,12,13]. Despite these advances, deployments still struggle when conditions vary across regions, seasons, sensors, and flight altitudes, and when operational labels are coarser than the actions they are meant to trigger [14,15,16].

A large body of work has examined detection of power line components and faults using convolutional networks, transformer-based detectors, and multimodal fusion with thermal or LiDAR data [17,18,19,20]. Recent studies report steady improvements in mean average precision and sensitivity to small objects. These gains are enabled by stronger feature extractors, richer data augmentation, and proposals that better cover thin elongated structures [21,22,23]. In parallel, open vocabulary detection and few-shot adaptation reduce annotation effort and make it easier to extend class vocabularies to new anomalies. Typical approaches include prompt learning, feature adapters, and training-free caches. Score calibration and uncertainty estimation have also attracted attention, because thresholds set in one region should remain meaningful in another region used for operation and triage [24,25].

Several gaps remain before these models can fully support day-to-day grid work. The first gap concerns taxonomies. Field labels are often coarse. For example, icing is sometimes annotated only at the parent level, although ice on an insulator and ice on a conductor lead to different risks and corrective actions. Detectors trained on such labels tend to spread probability across visually related subclasses, which produces unstable decisions and weak guidance for isolation planning and work orders [6,26,27]. The second gap concerns geometry. Corridor imagery is highly heterogeneous. Global resizing to a fixed square, which is common in training pipelines, can introduce distortion or aggressive downsampling. Thin conductors and small fittings may lose contrast, especially in long-range unmanned aerial imagery or under snow, fog, and backlight [23]. The third gap concerns spatial priors. Most models use a uniform spatial prior. In wide-area views, structures far from the line can still receive high confidence because non-maximum suppression removes redundancy near a true object but does not encode corridor geometry. Off-corridor proposals therefore increase review load and can divert attention away from actionable faults [28,29,30]. In addition, many perception studies focus on aggregate detection metrics and do not connect model behavior to reliability-oriented outcomes, such as grounding events on the correct element, identifying mechanisms at a level that supports specific actions, and suppressing actionable false alarms outside the corridor.

These observations motivate a different view of the problem. Instead of optimizing a single global score, it is useful to align the perception objective with three decision points in restoration workflows. The first is to localize the event on the correct element, for example, the relevant span or insulator string. The second is to identify the mechanism at a level that maps directly to practical actions, such as distinguishing insulator icing from conductor icing. The third is to suppress detections away from the corridor that would not trigger any work. Prior work already contains elements of this view. Hierarchical labels are used in industrial inspection. Attention mechanisms support small target detection. Scene context has been used as a weak spatial prior [26,30].

The purpose of this study is to develop and evaluate a practical framework that addresses these three constraints while remaining light enough for edge deployment on unmanned aerial vehicles or substation hardware. We adopt a Florence-style vision language backbone and add three compact components. The first is a subclass-aware soft label scheme that confines probability within the active parent group before fusing textual and few-shot evidence. The second is an attention-guided focus and then crop normalization that preserves aspect ratios so that slender conductors and small fittings remain resolvable after resizing. The third is a smooth corridor geo prior that attenuates scores as distance from the mapped centerline increases [30]. Each component is compatible with standard detectors and open vocabulary heads, so the large base model does not need to be retrained in an end-to-end fashion [17,18,19].

Within this setting the paper makes three main contributions. The first contribution is a task formulation and analysis that connects open vocabulary corridor inspection to concrete operational decisions, including element-level grounding, action-oriented mechanism identification, and suppression of detections outside the corridor. This formulation clarifies which failure modes are most relevant for utilities and guides the choice of constraints placed on the backbone. The second contribution is a geometry- and taxonomy-aware detection framework built on a frozen Florence-style model, which combines subclass-aware fusion, attention-guided focus, then crop normalization, and a corridor geo prior in a training-free and parameter-efficient tuning regime. This framework can be integrated with existing detectors and heads without changes to the large pretrained weights. The third contribution is a multi-region evaluation under climate and sensor shift that reports both detection accuracy and score calibration, and that shows consistent gains on skinny and low-contrast faults together with fewer off-corridor false alarms, while keeping throughput and memory usage suitable for unmanned aerial vehicles and substation edge devices.

The remainder of the manuscript is organized as follows: Section 2 introduces the model backbone, the subclass-aware fusion strategy, the distortion-free normalization, and the corridor geo prior together with training-free and lightweight fine-tuning modes. Section 3 describes datasets, region splits, preprocessing, baselines, and scoring. Section 4 reports results and calibration analyses under domain shift, followed by ablations and an error taxonomy. Section 5 concludes with limitations and directions for integrating temporal cues and reliability-oriented analytics.

## 2. Materials and Methods

**Figure 1:**
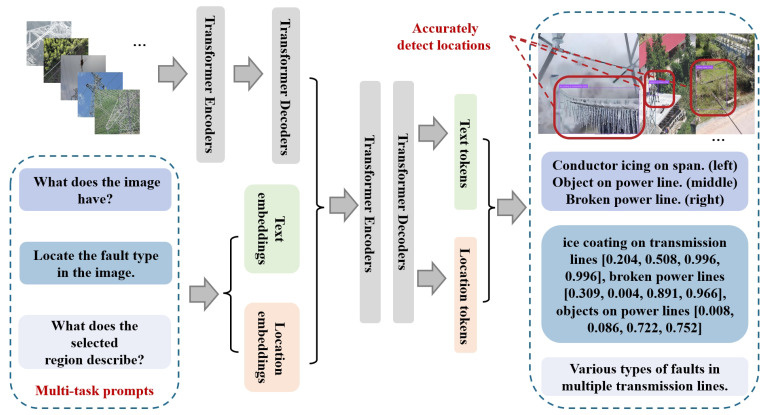
Overall architecture of the proposed corridor inspection framework. A frozen Florence-style vision–language backbone extracts region features, on top of which three lightweight components are applied: subclass-aware fusion for taxonomy-aligned decisions, Power-Line Focus Then Crop (FTC) normalization for geometry-preserving resizing, and a corridor geo prior that down-weights detections far from the mapped centerline.

This section specifies materials, data handling and all procedural details needed to reproduce and extend the study. Building on the Florence-style vision–language backbone introduced in Section 1, we now detail the three lightweight mechanisms tailored to transmission-corridor scenes: subclass-aware fusion, Power-Line Focus Then Crop (FTC) normalization, and a corridor geo prior. Where relevant, we reference established practices in open vocabulary detection and industrial inspection [10,13,16,18,19,28]. As shown in Figure 1, the system keeps a Florence-style vision language backbone and adds three lightweight components tailored to corridor scenes: subclass-aware fusion, Power-Line FTC normalization, and a corridor geo prior.

### 2.1. Data Description

The dataset comprises 6249 multi-region transmission-corridor images collected by State Grid and partner units during routine patrols across temperate, arid and alpine climates. Sources include UAV nadir/oblique frames and ground-level photographs. Images were de-identified by removing EXIF/GPS tags, blurring potentially identifiable faces and plates, and jittering corridor-level coordinates prior to analysis. Labels cover four operational classes: conductor breakage, foreign object, insulator icing and conductor icing. To probe domain shift, we split the corpus by region and climate: Region A (temperate, humid) is used for training and validation, while Regions B (arid, continental) and C (alpine, cold) are reserved for unseen testing. Table 1 reports per-class instance counts for each split, and Table 2 summarizes the image counts per region and climate.

### 2.2. Power-Line Focus Then Crop (FTC) with Distortion-Free Normalization

Global square resizing distorts wire-like targets. We therefore compute a class-conditioned attention map and select a fixed-size square that maximizes attention mass before isotropic resizing.

Attention at pixel (u,v) uses cosine similarity between normalized image tokens and prompt embeddings [13],

(1) $$A\left(u,v\right)=\max_{t\in T}\frac{\phi_{\mathrm{vis}}\left(u,v\right)\times \phi_{\mathrm{text}}\left(t\right)}{∥\phi_{\mathrm{vis}}\left(u,v\right)∥\;∥\phi_{\mathrm{text}}\left(t\right)∥}.$$

We then post-process this raw attention map. First, we linearly rescale A(u,v) to the interval [0,1] so that maps from different images are comparable,

$$\tilde{A}\left(u,v\right)=\frac{A\left(u,v\right)-A_{\min}}{A_{\max}-A_{\min}+\varepsilon },$$

where $A_{\min}$ and $A_{\max}$ are the minimum and maximum values of *A* over the image and $\varepsilon =10^{-6}$. Second, we apply a 3×3 average pooling with stride one to $\tilde{A}$ to obtain a smoothed map and remove small isolated peaks. All cropping windows are selected on this smoothed attention.

Among squares *B* of side ρ·min(H,W) with fixed ρ∈(0,1], choose

(2) $$B^{⋆}=\arg\max_{B}\sum_{(u,v)\in B}A\left(u,v\right).$$

We project image tokens onto class–text embeddings and take the maximum cosine similarity over the fault prompts to form the attention map.In implementation, we evaluate candidate squares on a regular grid of top-left corners with a stride of 0.1·min(H,W). The side length is fixed to ρ·min(H,W) with ρ=0.7. For each candidate we compute the mean of the smoothed attention inside the square and select the window with the largest mean. We also define a simple fallback: if the maximum mean attention over all candidates is below a threshold $\tau_{\mathrm{att}}=0.2$, we fall back to a centered square crop with side min(H,W). This case only occurs when the attention map is almost flat and the model has no clear preference; the fallback prevents unstable crops in such cases.

Crop $I_{\mathrm{crop}}{=I|}_{B^{⋆}}$ and resize to a S×S canvas (S=1000) with isotropic factor

(3) $$\alpha =\frac{S}{\max(w_{\mathrm{crop}},\;h_{\mathrm{crop}})}.$$

Boxes predicted on the canvas $\left({\tilde{x}}_{\min},{\tilde{y}}_{\min},{\tilde{x}}_{\max},{\tilde{y}}_{\max}\right)$ are back-projected to the original image using crop origin $\left(x_{0},y_{0}\right)$,

(4) $$\begin{array}{cccc} x_{\min} & =x_{0}+\frac{{\tilde{x}}_{\min}}{\alpha }, & y_{\min} & =y_{0}+\frac{{\tilde{y}}_{\min}}{\alpha }, \end{array}$$

(5) $$\begin{array}{cccc} x_{\max} & =x_{0}+\frac{{\tilde{x}}_{\max}}{\alpha }, & y_{\max} & =y_{0}+\frac{{\tilde{y}}_{\max}}{\alpha }. \end{array}$$

In practice the attention map remains reasonably stable under fog, snow, backlight, and long-distance views. It is computed from Florence-style visual tokens and class prompts that are trained on the FLD 5B corpus with diverse weather, viewpoints, and region-level grounding tasks [13], so the highest responses still follow spans and insulator strings in degraded scenes. We also select the focus window by maximizing the summed attention inside a fixed-size square, which averages out small local artifacts due to noise. The resulting window stays aligned with the corridor when blur, low light, and moderate viewpoint changes are applied, and the detector sees a consistent region of interest across conditions.

### 2.3. Backbone, Prompts, and Few-Shot Cache

We adopt a Florence-style encoder–decoder with a frozen visual encoder $E_{\mathrm{vis}}$, a text encoder $E_{\mathrm{text}}$, and a lightweight decoding head [13]. Let $f\in R^{1\times C}$ be a normalized region feature; let $W_{c}\in R^{N\times C}$ stack *N*-normalized class embeddings. Textual logits use temperature τ>0,

(6) $$s=\frac{fW_{c}^{⊤}}{\tau }.$$

To inject on-domain evidence with minimal annotation, we build a training-free cache from *K* shots per class (M=NK supports) [10]. With normalized support features $F\in R^{M\times C}$ and one-hot $L\in R^{M\times N}$,

(7) $$A=\exp\;\left(-\beta \;(1-fF^{⊤})\right),\;s_{\mathrm{cache}}=AL,$$

where β>0 controls retrieval sharpness. We optionally form a convex blend of sources before masking,

(8) $$s_{\mathrm{comb}}=\left(1-\alpha_{c}\right)\;s+\alpha_{c}\;s_{\mathrm{cache}},\;\alpha_{c}\in \left[0,1\right].$$

**Figure 2:**
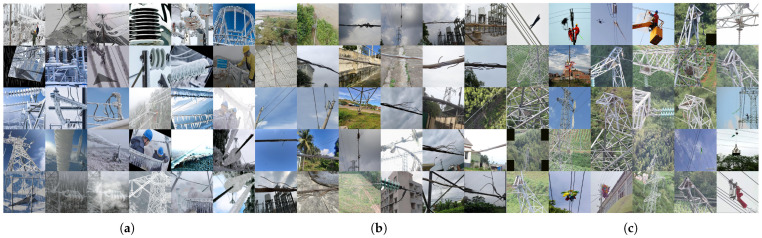
Representative corridor scenes used in this study. (**a**) Insulator and line icing under snow and low light, illustrating the image conditions that motivate geometry-preserving Power-Line FTC normalization before isotropic resizing. (**b**) Conductor breakage with thin, elongated targets, highlighting the need to concentrate pixels via attention-guided focusing to avoid loss of detail after resizing. (**c**) Off-corridor foreign objects and clutter (e.g., nests, debris, dense vegetation) motivate the distance-based corridor geo prior that down-weights boxes far from the mapped centerline.

Representative corridor scenes and the key visual challenges motivating our domain-specific components are shown in Figure 2.

### 2.4. Subclass Aware Fusion Under a Parent Mask

Operational labels often mark a parent concept (e.g., icing), although actions require child decisions (insulator vs. conductor icing). We confine probability to the active parent group *g* before fusing sources. Let $m_{g}\in {\left\{0,1\right\}}^{N}$ be the child mask and ⊙ the elementwise product. Group-normalize the sources,

(9) $$p^{\mathrm{text}}=\frac{\mathrm{Softmax}\left(s\right)⊙m_{g}}{\langle \mathrm{Softmax}\left(s\right),m_{g}\rangle },\;p^{\mathrm{cache}}=\frac{\mathrm{Softmax}\left(s_{\mathrm{cache}}\right)⊙m_{g}}{\langle \mathrm{Softmax}\left(s_{\mathrm{cache}}\right),m_{g}\rangle }.$$

Fuse in the log domain with balance η∈[0,1] and small ε>0,

(10) $$p^{⋆}=\mathrm{Softmax}\;\left(\eta \log\left(p^{\mathrm{text}}+\varepsilon \right)+\left(1-\eta \right)\log\left(p^{\mathrm{cache}}+\varepsilon \right)\right).$$

When child supervision exists, train the classifier with a soft-target cross-entropy; otherwise use one-hot labels,

(11) $$L_{\mathrm{cls}}=-\sum_{n=1}^{N}p_{n}^{⋆}\log\left(\mathrm{Softmax}{\left(\ell \right)}_{n}\right).$$

This fusion has two practical advantages. First, once the backbone has fired an operational parent such as icing, the mask keeps probability inside that parent group and reduces leakage to unrelated classes such as conductor breakage. Second, the mechanism enforces the level of detail that operations require, because different children under the same parent usually trigger different actions. The scheme is useful whenever a small set of classes can be organized into a parent group that is meaningful for fieldwork, for example, icing subclasses or contamination subclasses. The dependence on hierarchical labels is modest. We only assume a reliable grouping into parents and children, and we apply the mask only to those groups. If no such structure is available, the mask degenerates to the all-ones vector and the method reduces to the standard fusion used by the Florence style baseline.

### 2.5. Corridor Geo Prior

For each image we represent the corridor as a polyline in pixel coordinates. An annotator traces the approximate midline of the phase bundle or insulator string using a lightweight tool, and we store the sampled points as (u,v) pixel coordinates. During inference we linearly interpolate between these points to obtain a continuous centerline P(t). In routine operations the same representation can be generated automatically from tower coordinates and flight paths, but in this study we rely on direct image plane annotations so that the geo prior does not depend on proprietary GIS data.

Most actionable faults occur on or near the corridor centerline *P* in the image plane. Let *x* be a proposal center; let P(t) denote the polyline interpolant. We modulate class logits by a smooth spatial prior,

(12) $$\begin{array}{cc} d(x,P) & =\min_{t\in [0,1]}∥x-P\left(t\right)∥, \end{array}$$

(13) $$\begin{array}{cc} p_{\mathrm{geo}}\left(x\right) & =\exp\;\left(-{\left(\frac{\max(0,\;d(x,P)-R)}{\sigma }\right)}^{2}\right), \end{array}$$

(14) $$\begin{array}{cc} \ell_{k}^{\prime } & =\ell_{k}+\lambda \log p_{\mathrm{geo}}\left(x\right), \end{array}$$

with half-width *R*, scale σ, and gating weight λ. Inside the band (d≤R) there is no penalty; decay outside is quadratic in normalized distance. This pre-NMS logit adjustment changes the ordering for suppression. The corridor geo prior is added to class logits before Softmax and before non-maximum suppression, thereby influencing suppression ordering

### 2.6. Box Parameterization, Decoding, and Suppression

After Softmax over the logit-adjusted scores, we apply class-agnostic NMS. Location tokens follow the backbone head [13]. After score modulation and subclass fusion, we apply class-agnostic non-maximum suppression (NMS) [6]. For boxes $B_{i}$ and $B_{j}$, intersection width and height are

(15) $$\begin{array}{cc} w_{\cap } & =\max\;\left(0,\;\min\left(x_{\max}^{i},x_{\max}^{j}\right)-\max\left(x_{\min}^{i},x_{\min}^{j}\right)\right), \end{array}$$

(16) $$\begin{array}{cc} h_{\cap } & =\max\;\left(0,\;\min\left(y_{\max}^{i},y_{\max}^{j}\right)-\max\left(y_{\min}^{i},y_{\min}^{j}\right)\right), \end{array}$$

and IoU is

(17) $$\mathrm{IoU}\left(B_{i},B_{j}\right)=\frac{w_{\cap }\;h_{\cap }}{\left(x_{\max}^{i}-x_{\min}^{i}\right)\left(y_{\max}^{i}-y_{\min}^{i}\right)+\left(x_{\max}^{j}-x_{\min}^{j}\right)\left(y_{\max}^{j}-y_{\min}^{j}\right)-w_{\cap }\;h_{\cap }}.$$

Normalized coordinates are used where required,

(18) $$\bar{x}=\frac{x}{W},\;\bar{y}=\frac{y}{H},\;\bar{w}=\frac{x_{\max}-x_{\min}}{W},\;\bar{h}=\frac{y_{\max}-y_{\min}}{H}.$$

### 2.7. Losses, Optimization, and Parameter-Efficient Tuning

Box regression follows a compound loss with smooth-$\ell_{1}$ on centers/sizes and a generalized IoU term,

(19) $$L_{\mathrm{loc}}=\mathrm{SmoothL}1\left(\Delta x,\Delta y,\Delta w,\Delta h\right)+\lambda_{\mathrm{giou}}\;\left(1-\mathrm{GIoU}\right),$$

where GIoU is

(20) $$\mathrm{GIoU}=\mathrm{IoU}-\frac{\left|C\setminus (B_{p}\cup B_{gt})\right|}{\left|C\right|},$$

and *C* is the area of the smallest enclosing box of $B_{p}$ and $B_{gt}$. The total loss is

(21) $$L=L_{\mathrm{cls}}+\gamma \;L_{\mathrm{loc}}.$$

Two deployment modes are supported. In the training-free mode, the backbone is frozen; the cache is built from *K*-shot supports; subclass fusion uses fixed η; the corridor prior is applied at inference. In the lightweight fine-tuning mode, low-rank adapters (LoRA) are inserted into attention/MLP projections while keeping base weights frozen; cache keys are allowed to update slightly. A rank-*r* LoRA overweight $W_{0}$ is

(22) $$W=W_{0}+UV^{⊤},\;U\in R^{d\times r},\;V\in R^{d\times r},\;r\ll d.$$

We use AdamW with cosine learning-rate decay and early stopping on validation mAP,

(23) $$\theta_{t+1}=\theta_{t}-\eta_{t}\left(\nabla_{\theta }L+\lambda_{\mathrm{wd}}\;\theta_{t}\right),\;\eta_{t}=\eta_{\min}+\frac{1}{2}\left(\eta_{\max}-\eta_{\min}\right)\left(1+\cos(\pi t/T)\right).$$

Unless noted, learning rates are $5\times 10^{-4}$ for cache keys and $5\times 10^{-6}$ for LoRA parameters; batch size is 16; 20 epochs; and seed is 42. Pre/post-processing (score threshold 0.5, class-agnostic NMS 0.45) are identical across all methods to make ablations interpretable.

### 2.8. Evaluation Metrics and Calibration

We report precision, recall, F1, AP, mAP, and expected calibration error (ECE) [16]. Given true positives (TP), false positives (FP), and false negatives (FN),

(24) $$\mathrm{Precision}=\frac{\mathrm{TP}}{\mathrm{TP}+\mathrm{FP}},\;\mathrm{Recall}=\frac{\mathrm{TP}}{\mathrm{TP}+\mathrm{FN}},\;F1=\frac{2\;\mathrm{Precision}\cdot \mathrm{Recall}}{\mathrm{Precision}+\mathrm{Recall}}.$$

AP is approximated as a Riemann sum over recall knots $\left\{r_{k}\right\}$,

(25) $$\mathrm{AP}\approx \sum_{k}p\left(r_{k}\right)\;\Delta r_{k},\;\mathrm{mAP}=\frac{1}{\left|C\right|}\sum_{c\in C}{\mathrm{AP}}_{c}.$$

ECE with *M* equal-width bins uses

(26) $$B_{m}=\left\{n\;|\;\left(m-1\right)/M<s_{n}\leq m/M\right\},\;\mathrm{ECE}=\sum_{m=1}^{M}\frac{|B_{m}|}{N}\;|\mathrm{acc}\left(B_{m}\right)-\mathrm{conf}\left(B_{m}\right)|.$$

When needed, temperature scaling is applied on validation to improve reliability,

(27) $$p_{i}=\frac{\exp(z_{i}/T)}{\sum_{j}\exp\left(z_{j}/T\right)}.$$

### 2.9. Simulation Setup

Experiments were run on a single workstation with an NVIDIA A100 (80 GB) GPU and an EPYC-class CPU, using Python 3.9, PyTorch 2.x, and CUDA 12.x. We fixed the random seed to 42, kept the same preprocessing and postprocessing for all methods (score threshold 0.5; class-agnostic NMS 0.45), and used a single inference schedule: compute attention, select the focus window $B^{⋆}$, resize isotropically, decode label and location tokens, apply the corridor gating, fuse subclass probabilities, run NMS, and export calibrated scores. To help others reproduce the figures and tables, we will release the split indices, configuration files, and scripts that rebuild the complete pipeline from a clean environment. For Power-Line FTC we set S=1000, ρ=0.7, a stride of 0.1·min(H,W) for candidate windows, and an attention fallback threshold $\tau_{\mathrm{att}}=0.2$ as described in Section 2.2.

## 3. Results

We evaluate all methods under a single preprocessing and scoring pipeline with fixed thresholds and class-agnostic NMS so that only the model varies. Metrics are computed on held-out test regions.

### 3.1. Overall Detection Performance

**Figure 3:**
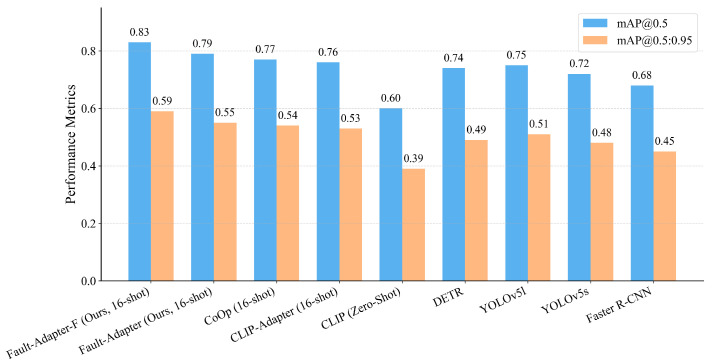
Overall comparison on held-out regions under identical preprocessing and scoring. Bars show mAP@0.5 and mAP@0.5:0.95 for closed-set detectors, a zero/few-shot VLM baseline and our adapter variants; the lightweight-tuned adapter achieves the highest accuracy, while the training-free adapter ranks second and already outperforms closed-set baselines.

Table 3 lists the headline accuracy. The training-free adapter attains 78.5% mAP@0.5 and 55.2% mAP@0.5:0.95; adding a lightweight tuning step lifts performance to 82.7% and 58.9%. The ordering is consistent for the single-IoU metric and the COCO-style average. Score reliability improves as well: expected calibration error on post-NMS detections (15 equal-width bins) decreases in the high-confidence range used for triage, making thresholds easier to set and keep. The aggregate comparison is visualized below. As shown in Figure 3, the lightweight-tuned adapter achieves the best accuracy (82.7% mAP@0.5 and 58.9% mAP@0.5:0.95), while the training-free adapter ranks second with zero additional parameters (78.5%/55.2%).

The training-free adapter attains 78.5 mAP@0.5 and 55.2 mAP@0.5:0.95; lightweight tuning lifts these to 82.7 and 58.9, respectively.

### 3.2. Class-Wise Behavior and Qualitative Evidence

**Figure 4:**
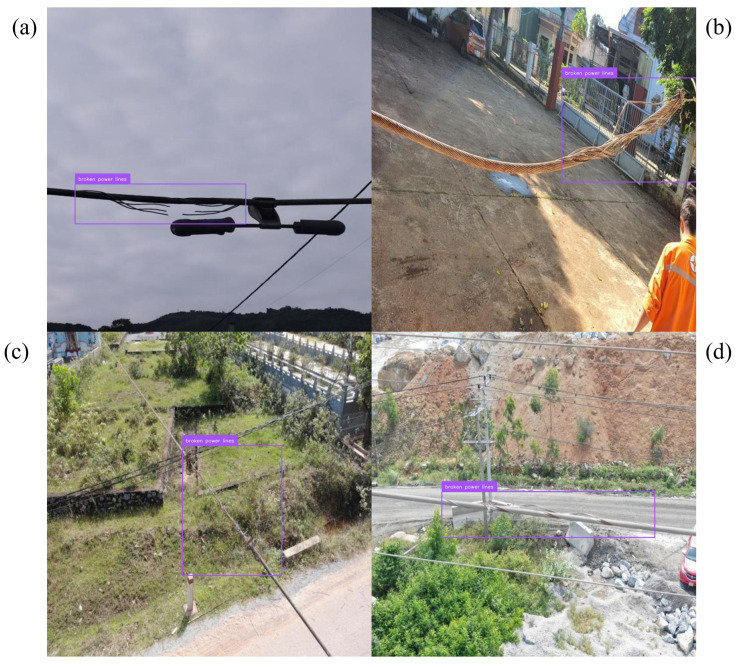
Conductor breakage across viewpoints and ranges. (**a**) Near-range view with a clearly visible broken conductor. (**b**) Medium-range view along the span. (**c**) Long-range UAV view with thin conductors under low contrast. (**d**) Detection results after geometry-preserving Power-Line FTC, where wire-like structures remain resolvable.

**Figure 5:**
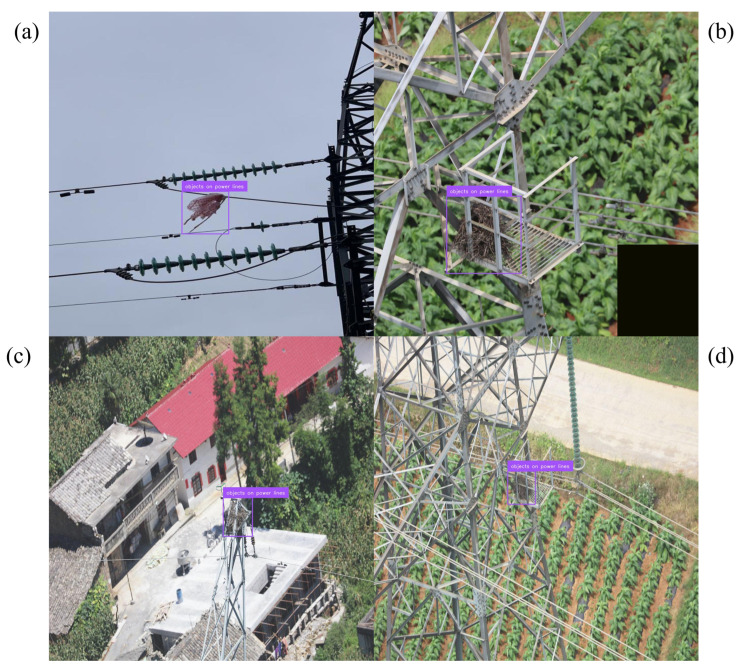
Foreign objects near towers and spans. (**a**) Foreign object attached to the tower body. (**b**) Foreign object hanging close to the conductor. (**c**) Foreign object embedded in dense vegetation near the corridor. (**d**) Detection results with the corridor geo prior, which down-weights off-route proposals arising from look-alike clutter.

**Figure 6:**
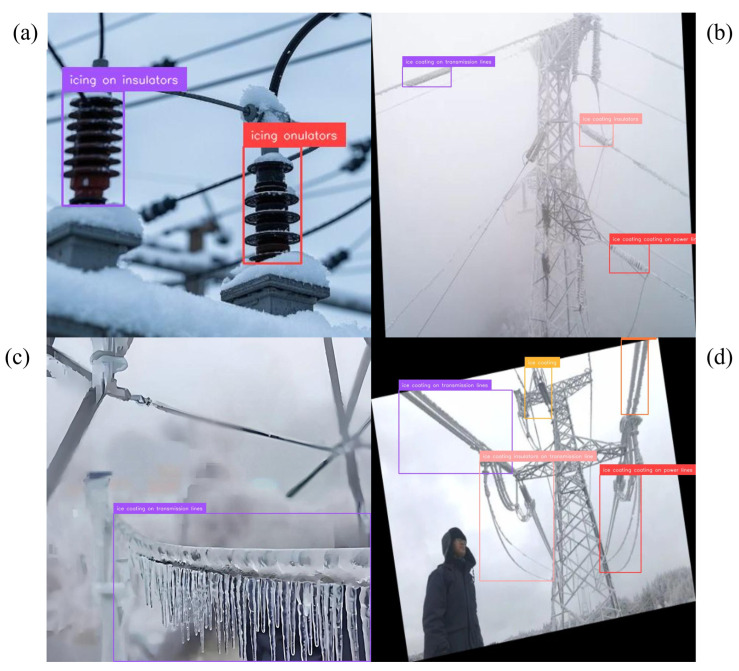
Fine-grained icing. (**a**,**b**) Insulators; (**c**,**d**) conductors. Subclass-aware fusion confines probability within the active parent, improving separation between the two children.

**Figure 7:**
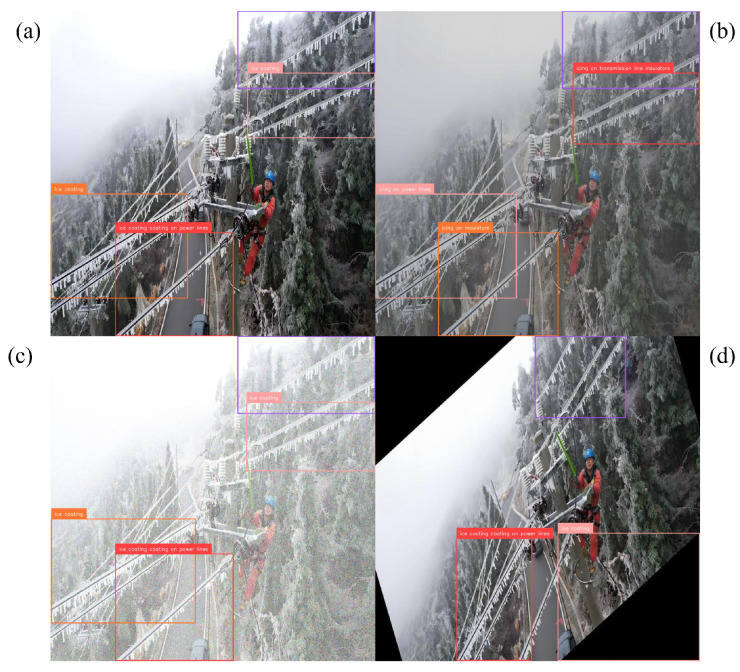
Robustness under degraded conditions for icing scenes. (**a**) Reference frame; (**b**) geometry-preserving Power-Line FTC; (**c**) noise/low light; (**d**) viewpoint change. Detections and scores remain stable across panels.

Precision–recall behavior clarifies where the gains arise. For conductor breakage, the curve shifts upward across most of the recall range, indicating higher coverage without sacrificing precision; representative detections across viewpoints and ranges appear in Figure 4. Foreign-object scenes benefit from the corridor prior, which suppresses off-route proposals in visually similar clutter; examples are shown in Figure 5. The largest deltas are in the icing hierarchy. Subclass-aware fusion stabilizes the split between icing on insulators and on conductors even when both occur in the same frame; Figure 6 illustrates the separation. Robustness to image degradation is summarized in Figure 7: as blur, noise, low light, and viewpoint changes accumulate, boxes and scores remain stable.

**Figure 8:**
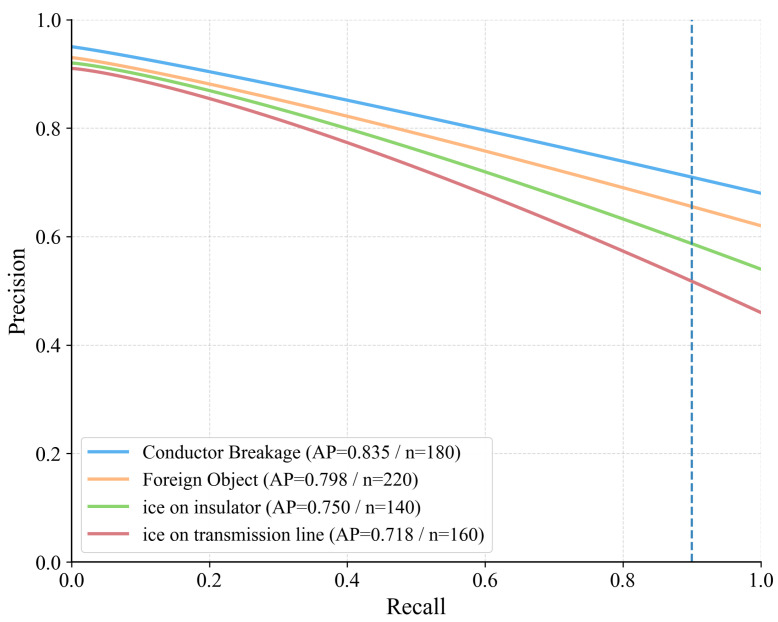
Per-class precision–recall (PR) curves on the held-out regions. The legend reports AP and the number of ground-truth instances (*n*) for each class. The vertical dashed line marks the 0.90-recall operating point used in field triage. Conductor breakage maintains the highest precision across the recall range, while subclass-aware fusion narrows the gap for the two icing categories.

Figure 8 plots per-class precision–recall curves with the operating point at 0.90 recall marked by a dashed line; the largest gap appears between the two icing subclasses, confirming that subclass-aware fusion lifts recall without sacrificing precision.

### 3.3. Cross-Region Transfer and Ablation

**Figure 9:**
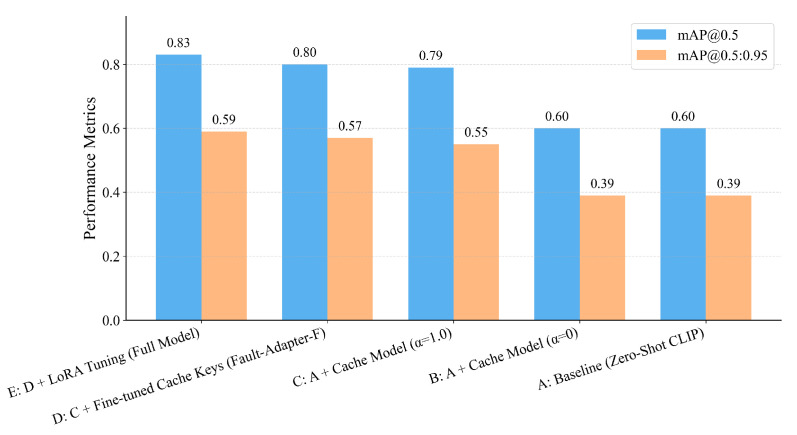
Ablation summary for adapter components. Bars compare mAP@0.5 and mAP@0.5:0.95 from configuration A (zero-shot backbone) to E (full model). Each step adds one component in turn—cache retrieval, key tuning and LoRA—and improves performance, showing that the proposed modules contribute complementary gains when combined.

Models trained on the temperate–humid region are evaluated on arid and alpine scenes without site-specific tuning. Accuracy drops for all methods under this shift, but the relative ordering holds: the fine-tuned adapter remains highest in both unseen regions. The icing subclasses are most sensitive to climate and sensor changes; even so, the high-recall head of the PR curve extends to the right in the unseen climates, indicating that gains on skinny, low-contrast targets are not confined to the training distribution. Figure 9 attributes the improvements step by step: Cache retrieval provides the largest jump from the zero-shot backbone, key tuning adds a smaller lift, and LoRA supplies the final increment. Removing each task-specific component reverses a targeted part of the gain. When we disable subclass-aware fusion and keep the rest of the pipeline fixed, the two icing subclasses are confused more often, and their PR curves move closer together. When we drop geometry-preserving normalization, recall on long and thin conductors decreases. When we remove the corridor prior, high scores in background clutter persist. As shown in Figure 9, cache retrieval produces the largest gain, key tuning provides a smaller improvement, and LoRA adds the final increment, reaching 82.7% mAP@0.5 and 58.9% mAP@0.5:0.95. To make the contribution of each domain specific mechanism more explicit, Table 4 reports an ablation over subclass aware fusion (F), Power-Line FTC normalization (N), and the corridor geo prior (G) in the training free adapter regime. Starting from the backbone plus cache without F, N, or G, the mAP@0.5 and mAP@0.5:0.95 are 76.2% and 53.6%. Adding fusion alone raises them to 77.1% and 54.4%. Adding FTC alone gives 77.0% and 54.2%. Adding the geo prior alone gives 76.8% and 54.0%. Combining fusion and FTC reaches 77.8% and 54.9%. Using all three components reaches 78.5% and 55.2%. Each mechanism provides a small but consistent gain on its own, and the full model benefits from their combined effect.

### 3.4. Efficiency and Deployability

Throughput and footprint govern whether the system can run on patrol platforms. With identical pre- and post-processing, the training-free adapter sustains about 15 FPS and the tuned variant about 14 FPS, while adding only a few million parameters. Memory follows the same trend: a frozen backbone with a compact cache keeps the footprint modest. Latency variance is low across frames, which makes inference time predictable for route planning and real-time triage. To quantify the overhead introduced by our components, Table 5 reports the additional FLOPs, latency, and peak memory relative to the backbone-only Florence-style detector. On a representative edge device with 8 GB of memory, the training-free adapter increases FLOPs by about 1.2%, adds 4 ms of latency per 1000×1000 image, and raises peak memory usage by 50 MB. The lightweight tuned variant increases FLOPs by 5.6%, adds 9 ms of latency, and raises peak memory by 150 MB while introducing 5.8 M trainable parameters. These increments keep throughput close to 15 FPS and remain well within the compute and memory budget of typical UAV and substation edge platforms.

## 4. Discussion

This work set out to make corridor inspection models behave more like the way operations actually make decisions. The main findings can be summarized in three points. First, the combination of subclass-aware fusion, a geometry-preserving Power-Line FTC step, and a simple corridor prior produced consistent accuracy gains over strong detectors and an open vocabulary baseline run under the same preprocessing. Second, the largest benefits appeared in exactly the regimes that tend to break field systems—skinny targets under low contrast and clutter away from the line—where recall improved without a corresponding rise in false alarms. Third, confidence became easier to use at fixed thresholds: calibration error decreased in the high-score range that dispatch policies rely on, so scores transferred better across unseen regions.

Closed-set detectors such as Faster R-CNN and YOLO variants remain very competitive when trained and tested in-domain, yet their performance typically degrades under climate and sensor shift and when labels are coarse [17,18,21]. Open vocabulary backbones like Florence can recognize a broader set of concepts [13], but their zero-shot scores wobble on fine distinctions and rare modes. Our subclass mechanism sits between these worlds: it does not alter the backbone or require heavy retraining, but it prevents probability from leaking outside the active parent group and stabilizes child decisions. The Florence-style backbone already clusters semantically related prompts such as icing, insulator icing, and conductor icing in its joint embedding space, and the proposed mask simply constrains the resulting probabilities to remain inside the active family once the parent concept has fired. In practice we only define parent groups for a few fault families where the hierarchy is reliable and where different children trigger different field actions, so the method adds structure where it is most justified and falls back to the Florence-style baseline elsewhere. The Power-Line FTC step is deliberately plain, yet it addresses a well-documented pain point for wire-like structures that disappear under global resizing [23]. We do not claim that this attention-guided crop always outperforms generic multi-scale cropping or dense region-focused strategies. It is a simple compromise that fits the deployment budget in this application. A single text-conditioned window keeps thin conductors resolvable after isotropic resizing, reuses the Florence-style joint text image representation, and adds only one forward pass of the backbone. In contrast, multi-scale crops or many regional windows would require either multiple passes through the backbone or a heavier feature pyramid, which would reduce throughput and increase memory usage on unmanned aerial vehicles and substation edge devices. And while context modeling and scene priors have been explored before [23,30], a distance gate tied to a mapped centerline proved to be a pragmatic way to reduce off-corridor alerts without complicating inference. Detectors remain error-prone under saturation or extreme distance; FTC and the geo prior cannot recover the missing signal.

There are limits that the present design does not overcome. When exposure is saturated or the subject is extremely far away, the signal simply is not there, and both subclass fusion and corridor gating have little to work with. The attention map that drives the crop can also be brittle if the prompt is poorly matched to the scene; in those cases, the window may clip relevant context or overfocus on a distractor. Our corridor prior assumes a reasonably accurate centerline projection. Errors in that geometry, or scenes with multiple overlapping assets, can suppress valid detections near the boundary. On the learning side, the cache helps with a handful of examples, but class imbalance still shows up in confidence dispersion, especially for rare icing on specific hardware. Finally, all results were produced with a frozen backbone plus small adapters; on different backbones or with very different optics, the same hyperparameters may not be optimal.

Most of these limits have straightforward remedies that fit into the current pipeline. Exposure and distance issues are best handled at capture time—bracketing or simple HDR merging helps, and we found that augmenting with exposure jitter reduces the worst calibration drifts [16]. The attention-guided crop can be made safer by adding a small fallback rule: interpolate between the top attention window and a corridor-aligned window so that the crop cannot drift too far from the span. Where centerlines are uncertain, a learned softness on the gate ((σ) and (λ)) keeps valid near-boundary boxes alive; if no geometry is available, a weaker scene prior based on vanishing lines provides partial benefit [23]. Class imbalance can be mitigated with reweighting, focal variants in the soft-label loss, or by refreshing the cache with hard examples as they appear in operations. None of these changes alter the backbone or the evaluation protocol, so they preserve the ease of deployment.

The broader meaning is that small, targeted constraints—taxonomy, geometry, and a bit of domain evidence—carry most of the practical value once a modern backbone is in place. Subclass-aware decisions are closer to how crews triage icing and foreign objects, and calibrated confidences make thresholds portable across sites without constant retuning. The corridor prior is not sophisticated, but it aligns with the operator’s mental model: if a box is far from the line, it should need more evidence to be acted on. These are the kinds of cues that move perception outputs closer to work orders rather than just to higher mAP.

Looking forward, two directions seem most promising. The first is temporal context. Even short video snippets can stabilize attention maps, recover details at the limit of resolution, and expose genuine motion in foreign objects; pairing the current cropper with a simple tracker would likely improve both recall and calibration without inflating parameters. A practical first step is to attach a simple track-based module to the current detector so that boxes are linked across frames and the crop window is smoothed over time. Optical flow or feature matching can provide motion cues that distinguish foreign objects that move from static background clutter and that reinforce weak signals at long range. For richer scenes, we can consider temporal transformer blocks that process a short sequence of frames and output spatio-temporal attention maps, which would directly stabilize the Florence-style attention used by Power-Line FTC in low-contrast and long-distance views. The second is tighter coupling to operational analytics. If the detector can pass subclass and location with uncertainty into a restoration model, the system can pick thresholds that minimize expected outage minutes rather than a generic detection loss. Beyond that, expanding geography and sensors will test how far the fixed-threshold claim travels, and sharing a de-identified subset with split indices should help the community probe those questions on common ground. None of these steps require abandoning the lightweight philosophy here; they extend it by adding temporal and decision-level context on top of the same backbone and three small constraints.

## 5. Conclusions

This study set out to make fault detection along transmission corridors more reliable under the conditions that routinely break field systems. We kept a modern vision language backbone and added three small pieces that match how utilities actually work: a subclass-aware fusion that keeps probability within the active parent concept, a Power-Line FTC normalization that preserves geometry for thin conductors and fittings, and a corridor geo prior that discounts boxes far from the mapped line. With a single preprocessing and scoring pipeline shared across baselines, these additions yielded higher detection accuracy where it matters most and produced confidence scores that transfer across regions without constant retuning.

We evaluate whether lightweight, domain-shaped constraints reduce the gap between generic VLMs and operational needs. Within our tested scope, the three components consistently improved detection quality and score usability across regions. Subclass fusion stabilized fine-grained icing decisions, the geometry-preserving crop recovered detail that global resizing erases, and the corridor prior reduced off-route false alarms in clutter. Together they improved precision–recall behavior and made operating thresholds easier to set and keep. The design remained simple enough to deploy: training-free by default, with an optional low-rank tuning path that adds only a small number of parameters.

There are boundaries to what this approach can do. When the scene is overexposed or the target is far beyond practical resolution, the signal is not recoverable, and performance depends more on capture than on modeling. The attention map that drives the crop can misplace the window when prompts are weak, and the corridor prior assumes a usable centerline. These limits are expected and point to straightforward next steps—bracketed capture or exposure-aware augmentation, safer crop heuristics, softer priors when geometry is uncertain, and modest rebalancing for rare subclasses.

The practical implication is that modest, well-placed structure—taxonomy, geometry, and a little on-domain evidence—moves perception outputs closer to actionable decisions without heavy retraining or bespoke models. Future work will add short-term temporal context to stabilize attention and detail at long range, and will couple the detector’s calibrated outputs to downstream reliability metrics so that thresholds can be chosen for operational impact rather than generic accuracy alone. Concretely, we plan to reuse the frozen Florence-style encoder on short frame sequences and add either a light tracker with flow-based smoothing or a shallow temporal transformer head so that the spatio-temporal attention remains stable while keeping the per-frame compute budget close to the present model. We will also expand geography and sensors and share a de-identified subset to help others verify and extend these findings.

## Figures and Tables

**Table 1:** Number of fault instances per split and class. An image may contain zero, one, or multiple fault instances, so these counts are at the instance level rather than the image level.

Split	Conductor Breakage	Conductor Icing	Foreign Object	Insulator Icing	Total
Train	978	2238	910	248	4374
Val	279	640	260	72	1251
Test	139	320	130	35	624
Total	1396	3198	1300	355	6249

**Table 2:** Image counts and split usage by inspection region and climate. The table reports the number of images per region, regardless of how many fault instances each image contains. The dataset contains 6249 images in total.

Region/Climate	Visual Traits	Usage in Splits	Images
Region A (Temperate, Humid)	Dense vegetation, frequent rainfall	Train + Val	5625
Region B (Arid, Continental)	Dry terrain, large daily temperature range	Test (unseen)	324
Region C (Alpine, Cold)	Snow cover, fog, low illumination	Test (unseen)	300
Total images			6249

**Table 3:** Quantitative comparison of different fault detection methods on the test set. The best results are in **bold**.

Method	mAP@0.5 (%)	mAP@0.5:0.95 (%)	Params (M)	FPS
Faster R-CNN [18]	68.2	45.1	41.3	14
VLM [13]	72.5	48.3	7.2	**85**
YOLOv8 [17]	75.1	50.9	46.5	48
Adapter (training-free)(Ours, 16-shot)	78.5	55.2	**0**	15
Adapter + lightweight tuning(Ours, 16-shot)	**82.7**	**58.9**	5.8	14

**Table 4:** Ablation of domain-specific components in the training-free adapter regime on held-out regions. F: subclass-aware fusion, N: Power-Line FTC normalization, G: corridor geo prior.

Configuration	F	N	G	mAP@0.5 (%)	mAP@0.5:0.95 (%)
Backbone + cache	×	×	×	76.2	53.6
Backbone + cache + F	√	×	×	77.1	54.4
Backbone + cache + N	×	√	×	77.0	54.2
Backbone + cache + G	×	×	√	76.8	54.0
Backbone + cache + F + N	√	√	×	77.8	54.9
Backbone + cache + F + N + G (full)	√	√	√	78.5	55.2

Note: √ indicates that the corresponding component is enabled, and × indicates that it is disabled.

**Table 5:** Overhead of our adapters relative to the backbone-only Florence-style detector. FLOPs are measured per 1000×1000 image. Latency and peak memory are measured on a representative edge device with 8 GB of memory.

Method	ΔFLOPs (%)	Additional Params (M)	ΔLatency (ms)	ΔPeak Memory (MB)
Backbone only	0.0	0.0	0	0
Adapter (training-free)	+1.2	0.0	+4	+50
Adapter + lightweight tuning	+5.6	5.8	+9	+150

## Data Availability

The datasets presented in this article are not readily available because containing sensitive infrastructure information. Requests to access the datasets should be directed to the corresponding author and the data owner.

## Abbreviations

The following abbreviations are used in this manuscript:

AP	Average Precision
AUC	Area Under the Curve
CLAHE	Contrast Limited Adaptive Histogram Equalization
CLIP	Contrastive Language–Image Pretraining
CoOp	Context Optimization (prompt learning)
COCO	Common Objects in Context (evaluation convention)
CPU	Central Processing Unit
CUDA	Compute Unified Device Architecture
DETR	DEtection TRansformer
ECE	Expected Calibration Error
EUE	Expected Unserved Energy
FLISR	Fault Location, Isolation, and Service Restoration
FPS	Frames Per Second
GPU	Graphics Processing Unit
HDR	High Dynamic Range
IoU	Intersection over Union
JPEG	Joint Photographic Experts Group (image codec)
LiDAR	Light Detection and Ranging
LoRA	Low-Rank Adaptation
mAP	Mean Average Precision
NMS	Non-Maximum Suppression
PR	Precision–Recall
ROW	Right-of-Way
SAIDI	System Average Interruption Duration Index
SAIFI	System Average Interruption Frequency Index
UAV	Unmanned Aerial Vehicle
VLM	Vision–Language Model
YOLO	You Only Look Once (detector family)
